# Predicting the deltoid tuberosity index in proximal humerus fractures using fracture characteristics and patient age: development of the LBQ-PHF score

**DOI:** 10.1186/s12891-023-06883-z

**Published:** 2023-09-25

**Authors:** Sam Razaeian, Okba Al Marhi, Birgitt Wiese, Dafang Zhang, Panagiotis Bouklas, Christian Krettek, Nael Hawi

**Affiliations:** 1 Department of Trauma Surgery, Hannover Medical School, Carl-Neuberg-Str. 1, 30625 Hannover, Germany; 2 Department of Trauma Surgery and Orthopaedic Surgery, Helios St. Marienberg Klinik Helmstedt GmbH, Conringstraße 26, 38350 Helmstedt, Germany; 3 Hannover Medical School, MHH Information Technology (MIT), Carl-Neuberg-Str. 1, 30625 Hannover, Germany; 4 Department of Orthopaedic Surgery, Brigham and Women’s Hospital, 75 Francis St, Boston, MA 02115 USA; 5 Hannover Humerus Registry (HHR), Traumastiftung gGmbH Carl-Neuberg-Str. 1, 30625 Hannover, Germany; 6 Orthopaedic and Surgical Clinic Braunschweig (OCP), Mauernstraße 35, 38100 Braunschweig, Germany

**Keywords:** Proximal humerus fracture, Local bone quality, Deltoid tuberosity index, Fracture morphology, Fracture pattern, Valgus, Score

## Abstract

**Background:**

The aim of this study was to investigate (1) whether fracture pattern and age are associated with local bone quality (LBQ), and (2) whether a scoring system based on these variables is able to predict LBQ in proximal humerus fractures (PHF).

**Materials and methods:**

A retrospective study was performed of all acute PHF at a Level 2 trauma center with plain radiographs and CT between June 2009 and March 2022. Local bone quality was measured by using the deltoid tuberosity index (DTI). In addition to age and gender, fracture morphology was categorized using the following classification systems: Neer, Resch, AO Foundation/Orthopaedic Trauma Association (AO/OTA), and Hertel/LEGO. Additionally, coronal head alignment was calculated by measuring the head-shaft angle.

**Results:**

Only the Resch classification system revealed a significant relationship between fracture type and bone quality, as there was a significant association between coronal head alignment and DTI (p = 0.001). Valgus head alignment was observed significantly more frequent in patients with low bone quality (p = 0.002). Multinomial logistic regression analysis revealed a significant relative risk ratio for age (RRR = 0.97, [95% CI, 0.94-1], p = 0.039) and a non-significant trend for DTI (RRR = 1.26, [95% CI, 0.96–1.64], p = 0.092) for occurrence of anatomic relative to valgus head alignment. Using a DTI cut-off value of 1.3 instead of 1.4, age and also varus head alignment were identified as significant predictors of LBQ (OR = 1.12, [95% CI, 1.1–1.15], p < 0.001; OR = 0.54, [95% CI, 0.3–0.96], p = 0.037). A scoring system called the LBQ-PHF score (local bone quality in proximal humerus fractures), developed based on these two variables was able to predict LBQ with a sensitivity of 79.2% and a specificity of 86.7%.

**Conclusion:**

Age and coronal humeral head alignment are independent predictors of LBQ in PHF. A simple scoring system developed based on these variables is able to assess BQ with solid predictive characteristics.

## Introduction

Several studies have shown that local bone quality (LBQ) may affect surgical outcomes and functions as a predictor for osteosynthesis failure [1–3].

Therefore, some authors have concluded that LBQ should be part of the preoperative assessment and have included this variable in recently recommended treatment algorithms [4].

To measure LBQ of PHF, the deltoid tuberosity index (DTI) was developed, which is directly measured proximal to the deltoid tuberosity on the anteroposterior plain radiograph [5, 6]. This index has been shown to strongly correlate with the bone mineral density of the humeral head as measured with peripheral quantitative computed tomography (pQCT) [6]. The DTI is so far the only validated radiographic measurement for bone mineral density (BMD) in PHF [6]. Only a few studies to date with rather small data sets and limited usage of fracture classification systems have dealt with the relationship between LBQ measured using this index and fracture morphology [5, 7, 8].

The aim of this study was to investigate using a large data set and multiple fracture classification systems (1) whether age and fracture pattern, in particular coronal head alignment, are associated with local bone quality, and (2) whether a scoring system based on these variables is able to predict LBQ in PHF.

.

## Materials and methods

The aim and motivation for this investigation are based on so far unpublished observations of the first and last author (S. R. and N. H.) from a prospective, CT-based single-center observational registry study of a supraregional Level 1 trauma center (Hannover Humerus Registry – HHR, NCT03060876). An association between age, coronal head alignment, and local bone quality (LBQ) in PHF was suggested. Gender was not assumed to have any association with LBQ. These assumptions were based on observations made using the Resch classification system, which relies on descriptive fracture morphology, including coronal humeral head alignment. This observation shall be reviewed on external data by one independent observer.

### Patients

**Fig. 1:**
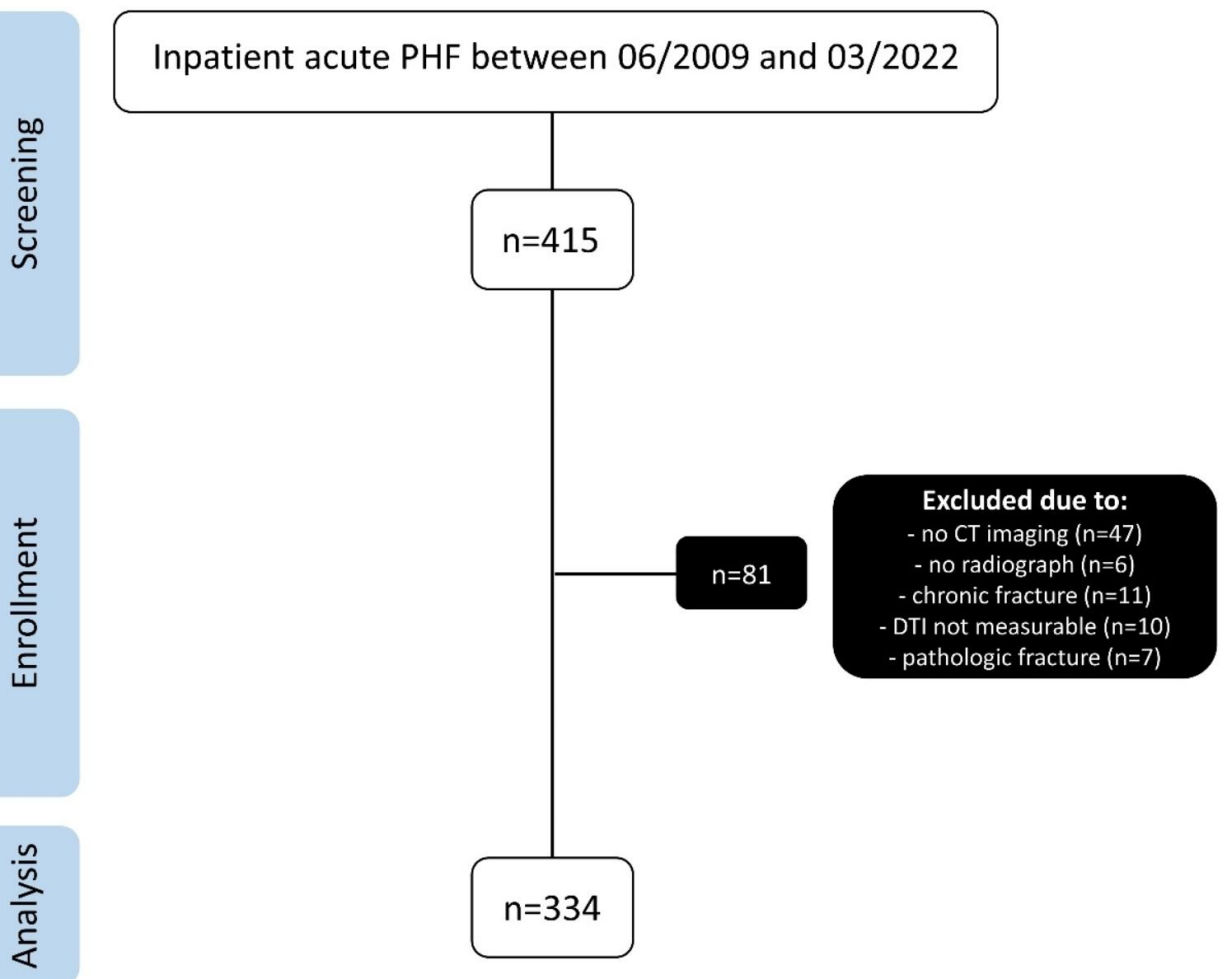
Flow chart of study inclusion. Acute fractures were defined as PHFs under 6 weeks after injury

A retrospective study was performed of all inpatient cases of acute PHF (under 6 weeks after injury) at a Level 2 trauma center with complete radiographic imaging (plain radiographs and CT) between June 2009 and March 2022. Patients were identified by searching in the institutional database for coded diagnosis (S42.20-24) according to the International Classification of Diseases − 10th Revision. Figure 1 illustrates details of study inclusion using a flow chart.

This study was approved by the local ethics committee (Ärztekammer Niedersachsen) and carried out in accordance with the ethical standards of the 1964 Declaration of Helsinki as updated in 2004. Only those patients were retrospectively screened and included that gave written informed consent to data usage for research purposes.

### Imaging analyses

DTI was determined on anteroposterior (AP) plain radiographic images as described by Spross et al. with picture archiving and communication system (PACS) by one senior orthopaedic trauma surgeon (O. A.), who was blinded to the study hypothesis. DTI was also categorized into two groups according to the cutoff recommended for local LBQ in PHF by Spross et al., lower than 1.4 and greater than or equal to 1.4 [6].

All fractures were classified by the same observer using both imaging modalities (plain radiographs and 2-D CT) according to the classification systems by Neer, Resch, the AO Foundation/Orthopaedic Trauma Association (AO/OTA) system of 2018, and the binary (LEGO) description system of the Hertel classification [9–13]. Additionally, coronal head alignment was calculated with a threshold of 10°. For this purpose, the head-shaft angle (HSA) was measured using plain radiography as described by Agudelo et al. [14]. As an anatomic HSA was defined as 135°, values greater than 145° and lower than 125° were defined as valgus and varus displacement, respectively. Isolated tuberosity fractures were defined as “anatomic”. Variables including age and gender were also collected as potential explanatory variables.

### Statistical analyses

Descriptive statistics were calculated. Chi-squared statistics were used to detect any association between gender, DTI, and fracture pattern. If cells had expected frequencies less than 5, then Fisher’s exact test was used. For post-hoc test in case of significant analysis, Bonferroni correction was used.

In order to investigate the potential influence of independent variables on the coronal head alignment with “valgus” as reference group, multinomial logistic regression analysis was performed to calculate relative risk ratios (RRR). For continuous potential predictor variables of age and DTI, reference unit changes of “1” and “0.1” were defined. Subsequent logistic regression analysis was performed to identify potential predictors (age, gender, and coronal head alignment with “valgus” as reference group) of LBQ. For this purpose, DTI as the target variable was grouped according to the most optimal cut-off value calculated through classification and regression tree (CART) modeling analysis.

For bivariate analysis of correlation between numeric factors, the Pearson correlation coefficient was calculated. Correlation strength was classified as follows: very high, r > 0.90; high, r = 0.70–0.89; moderate, r = 0.50–0.69; fair, r = 0.30–0.49; low, r = 0.10–0.29; or very low, r < 0.10. Data analysis was performed by one independent senior biometrician with SPSS 26.0 (IBM, Armonk, New York), STATA 16.1 (StataCorp), and R Statistical Software including rpart package (version 4.1.19; R Foundation for Statistical Computing, Vienna, Austria). The confidence level was set at 95% (p < 0.05).

## Results

**Fig. 2:**
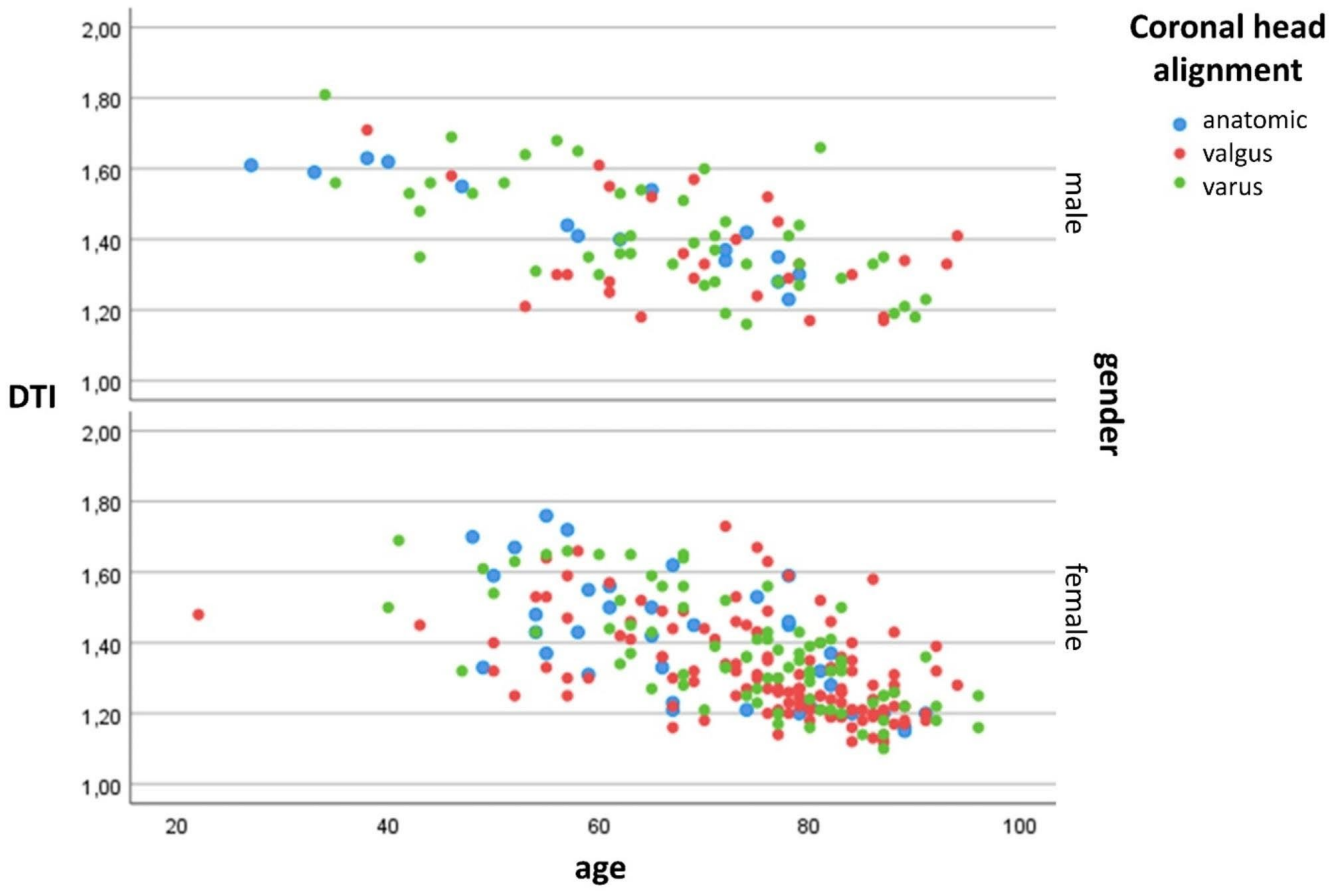
Graphical relationships between age, DTI, coronal head alignment, and gender

Three hundred thirty-four acute PHF were analyzed. Table 1 shows the distribution of age, gender, DTI, and fracture pattern. Figure 2 shows the graphical relationship between age, DTI, coronal head alignment, and gender. In both genders, male and female, age significantly correlated with DTI (r = -0.63, and r = -0.6, p = 0.01).

**Table 1:** Distribution of age, gender, and fracture pattern in relation to DTI is shown. ^a^Mann-Whitney U test with two-sided level of significance (α = 0.05). ^b^Chi-squared test with asymptomatic two-sided level of significance (α = 0.05). Bold values indicate significant values after Bonferroni correction as a post-hoc test following chi-square analysis (corrected levels of significance: ^c^α = 0.004, ^d^α = 0.003, ^e^α = 0.004, ^f^α = 0.008)

		DTI		
		< 1.4	≥ 1.4	p-value
**Age in years** mean (range) ± SD	72.04 (22–96) ± 13.62	77.5 (43–96) ± 10.2	62.8 (22–94) ± 13.7	0.001^a^
**Gender** n (%)				0.034^b^
female	244 (73.1)	161 (66)	83 (43)	
male	90 (26.9)	48 (53.3)	42 (46.7)	
**Resch classification** n (%)				0.001^b^
1	47 (14.07)	21 (44.7)	26 (55.3)	0.006^c^
2	27 (8.08)	20 (74.1)	7 (25.9)	0.2^c^
3	140 (41.92)	101 (72.1)	39 (27.9)	**0.002** ^**c**^
4	103 (30.84)	60 (58.3)	43 (41.7)	0.28^c^
Anterior fracture dislocation	16 (4.79)	6 (37.5)	10 (62.5)	0.03^c^
Posterior fracture dislocation	1 (0.3)	1 (100)	0 (0)	0.44^c^
**AO/OTA classification** n (%)				0.78^b^
A	125 (37.43)	81 (64.8)	44 (35.2)	
B	116 (34.73)	72 (62.1)	44 (37.9)	
C	93 (27.84)	56 (30.2)	37 (39.8)	
**Hertel/Lego classification** n (%)				0.025^b^
1	95 (28.4)	69 (62.6)	26 (27.4)	0.017^d^
2	2 (0.6)	2 (100)	0 (0)	0.27^d^
3	27 (8.1)	10 (37)	17 (63)	0.004^d^
4	1 (0.3)	0 (0)	0 (100)	0.2^d^
5	1 (0.3)	1 (100)	0 (0)	0.44^d^
6	0 (0)	0 (0)	0 (0)	
7	117 (35)	73 (62.4)	44 (37.6)	0.96^d^
8	7 (2.1)	5 (71.4)	2 (28.6)	0.62^d^
9	6 (1.8)	4 (66.7)	2 (33.3)	0.83^d^
10	0 (0)	0 (0)	0 (0)	
11	0 (0)	0 (0)	0 (0)	
12	78 (23.4)	45 (57.7)	33 (43.3)	0.31^d^
**Neer classification** n (%)				0.031^b^
1-Part	40 (12)	19 (47.5)	21 (52.5)	0.036^e^
2-Part	146 (43.7)	101 (69.2)	45 (30.8)	0.028^e^
3-Part	84 (25.1)	53 (63.1)	31 (36.9)	0.91^e^
4-Part	32 (9.6)	19 (59.4)	13 (40.6)	0.69^e^
Anterior fracture dislocation	19 (5.7)	7 (36.8)	12 (63.2)	0.018^e^
Posterior fracture dislocation	1 (0.3)	1 (100)	0 (0)	0.44^e^
Articular surface/Head split	12 (3.6)	9 (75)	3 (25)	0.37^e^
**Coronal head alignment** n (%)				0.001^b^
anatomic	52 (15.57)	23 (42.2)	29 (55.8)	**0.003** ^f^
valgus	157 (47)	112 (71.3)	45 (28.7)	**0.002** ^f^
varus	125 (37.43)	74 (59.2)	51 (40.28)	0.32^f^

Among all classification systems, only the morphological Resch classification system revealed a significant relation between fracture type and bone quality as there was a significant association between coronal head alignment and DTI (p = 0.001) (Table 1). Valgus head alignment was observed significantly more frequent in patients with low bone quality (p = 0.002) (Table 1). While fracture severity according to the AO/OTA classification system was not associated with bone quality, nonsignificant trends were observed in fracture type distribution according to the Neer and Hertel/Lego classification systems. Displaced 2-, 3-, and 4-part fractures, and Hertel/Lego type 1 fractures more frequently had LBQ.

### Classification and regression tree (CART) modeling analysis

**Fig. 3:**
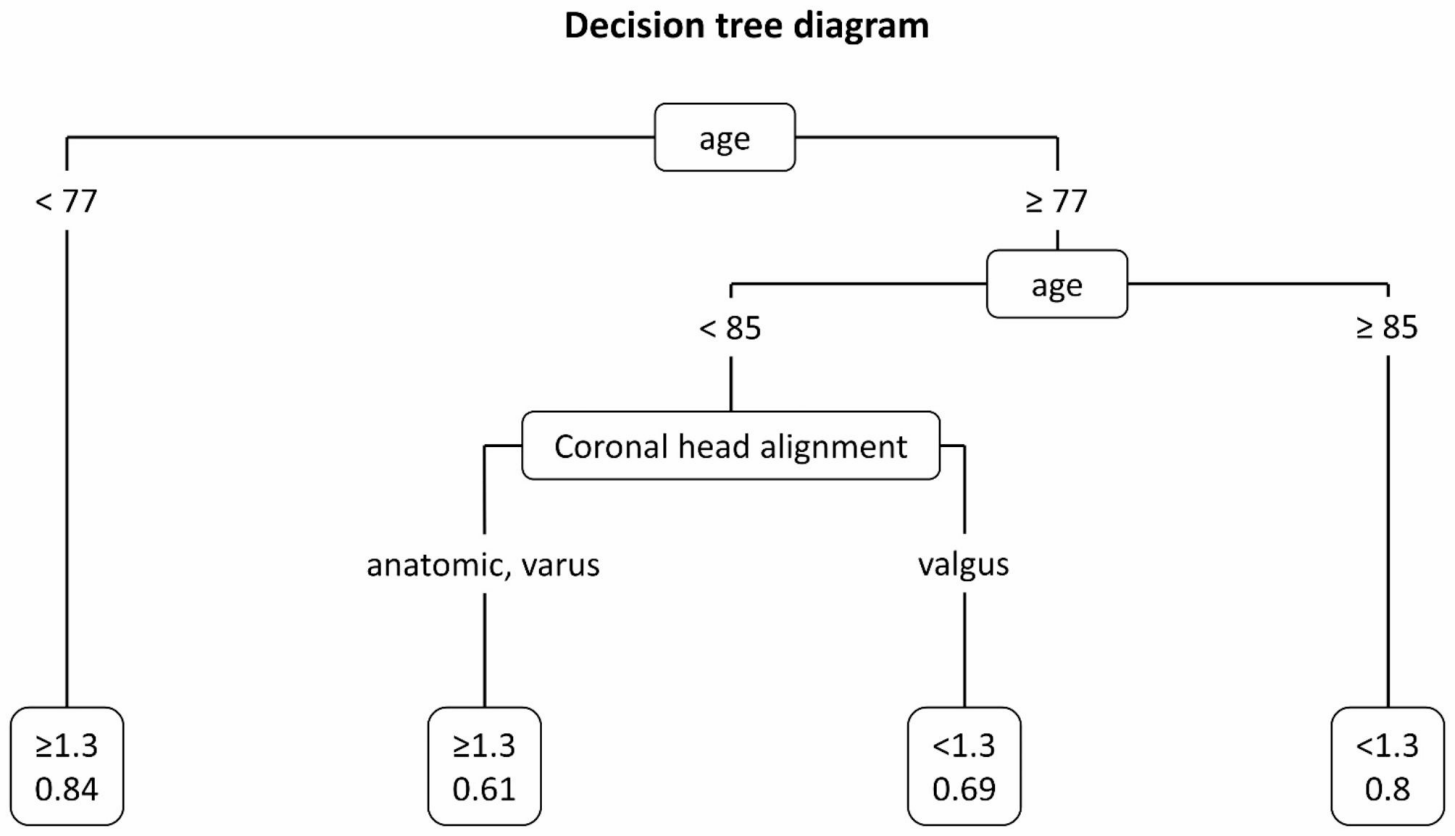
Decision tree diagram through classification and regression tree (CART) modeling analysis

CART modeling analysis revealed a DTI of < 1.3 as an optimal cutoff value for poor LBQ as the outcome variable. The decision tree diagram as a result of this analysis shows the optimal dividing points for age and coronal head alignment, while gender could not be identified as an appropriate predictor (Fig. 3). Table 2 shows frequency distribution of the individual groups.

**Table 2:** Frequency distribution of the individual groups of CART modeling analysis

	DTI		
	< 1.3	≥ 1.3	Total
Age < 77	29	151	180
	16.1%	83.9%	
Age 77–84 and anatomic or varus	17	27	44
	38.6%	61.4%	
Age 77–84 and valgus	37	17	54
	68.5%	31.5%	
Age ≥ 85	45	11	56
	80.4%	19.6%	
Total	128	206	334
	38.3%	61.7%	

### Logistic regression analysis

Multinomial logistic regression analysis revealed a significant relative risk ratio for age (RRR = 0.97, [95% CI, 0.94-1], p = 0.039) and a non-significant trend for DTI (RRR = 1.27, [95% CI, 0.96–1.64], p = 0.092) for the occurrence of anatomic relative to valgus head alignment. In addition, male gender and DTI were significant relative risk factors for the occurrence of varus relative to valgus head alignment (RRR = 2.4, [95% CI, 1.37–4.23], p = 0.002, and RRR = 1.27, [95% CI, 1.03–1.56], p = 0.026). Subsequent logistic regression analysis revealed age, but not head alignment, as a highly significant independent predictor of LBQ (OR = 1.1, [95% CI, 1.1–1.13], p < 0.001). Using a DTI cut-off value of 1.3 instead of 1.4, age and also varus head alignment were identified as significant predictor of LBQ (OR = 1.12, [95% CI, 1.1–1.15], p < 0.001; OR = 0.54, [95% CI, 0.3–0.96], p = 0.037).

### Development of a scoring system to predict local bone quality

The basis for scoring system development is a logistic regression in which the outcome variable is good or poor bone quality (DTI > = 1.3 or < 1.3, respectively). Typically, one would divide the cohort into two parts, using one subcohort of subjects to develop the score and the other subcohort of subjects to validate the score. However, since the cohort was not that large, the total number of cases was used for scoring system development, and validation was performed on an external unmatched test cohort of 107 consecutive cases from the abovementioned observational registry study.

For simplicity of designing the scoring system, age in years was categorized as follows with corresponding frequencies (n [%]): < 65 (89 [26.6]), 65–69 (35 [10.5]), 70–74 (35 [10.5]), 75–79 (62 [18.6]), 80–84 (57 [17.1]), ≥ 85 (56 [16.8]). In addition, for coronal alignment, the anatomic and varus alignment groups were merged. Results of logistic regression analysis with these age groups, gender, and coronal alignment parameters are shown in Table 3. All parameters except gender showed significant influence. In order to verify whether there was an improvement in the prediction model with a parameter with less influence removed, the Akaike’s information criterion (AIC) and Bayesian information criterion (BIC) were calculated. These showed better predictive model characteristics without gender as a parameter (Table 4).

**Table 3:** Logistic regression analysis revealed that all parameters except gender showed significant influence. Akaike’s information criterion and Bayesian information criterion for this model were 352.4 and 382.88

Age in years	Odds ratio	Standard error	P>|z|	95% Confidence Interval
65–69	3.87	2.28	0.022	1.22–12.3
70–74	5.39	3.1	0.003	1.77–16.43
75–79	9.47	4.74	< 0.001	3.55–25.26
80–84	14.88	7.64	< 0.001	5.43–40.71
≥ 85	50.62	27.62	< 0.001	17.37–147.51
**Coronal head alignment**				
valgus	1.82	0.49	0.028	1.07–3.01
**Gender**				
female	1.2	0.41	0.59	0.62–2.33

**Table 4:** Logistic regression analysis without gender as a parameter. Akaike’s information criterion and Bayesian information criterion for this model were 350.68 and 377.36

Age in years	Odds ratio	Standard error	P>|z|	95% Confidence Interval
65–69	4	2.35	0.018	1.26–12.63
70–74	5.43	3.08	0.003	1.78–16.51
75–79	9.76	4.86	< 0.001	3.68–25.89
80–84	15.75	7.93	< 0.001	5.87–42.62
≥ 85	52.34	28.42	< 0.001	18.06–151.69
**Coronal head alignment**				
valgus	1.86	0.5	0.021	1.1–3.15

**Fig. 4:**
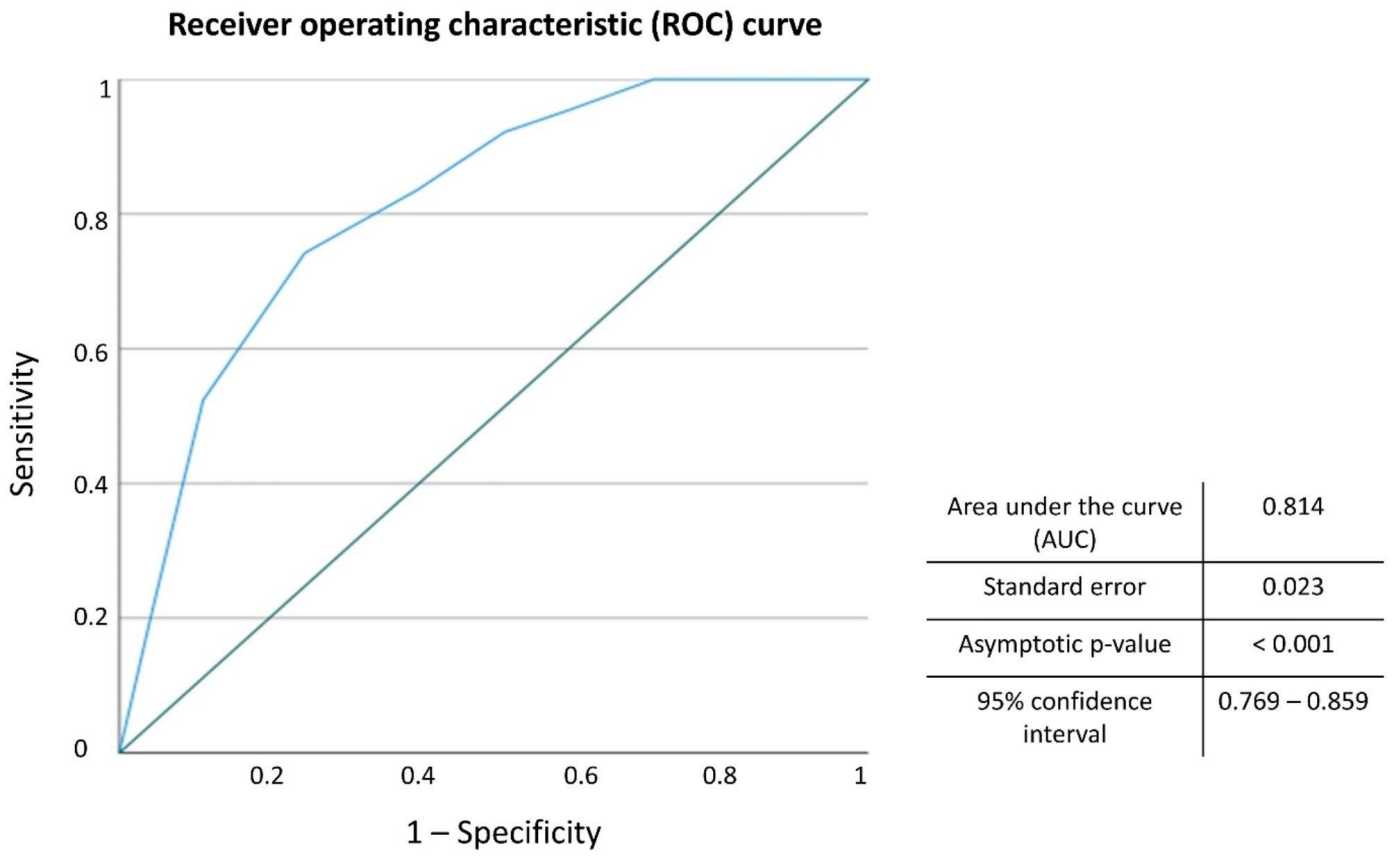
Receiver operating characteristic (ROC) curve analysis of the LBQ-PHF scoring system

To develop a scoring system, the regression coefficients were used. In order to have more usable regression coefficients, these were converted to integer points by dividing them by the lowest coefficient and rounding. This scoring system called the LBQ-PHF (local bone quality in proximal humerus fractures) score and the corresponding receiver operating characteristic (ROC) curve are shown in Table 5; Fig. 4.

**Table 5:** The LBQ-PHF scoring system. The points are summated. A maximum of 7 points can be achieved. *Converted regression coefficients are calculated by dividing the regression coefficient by the lowest coefficient

Age in years	Regression coefficient	Converted regression coefficient*	Points
< 65			0
65–69	1.39	2.2	2
70–74	1.69	2.7	3
75–79	2.28	3.7	4
80–84	2.76	4.5	5
≥ 85	3.96	6.4	6
**Coronal head alignment**			
anatomic/varus			0
valgus	0.62	1	1

### Determination of a threshold value for the LBQ-PHF score and external validation

To determine an appropriate threshold value for the total LBQ-PHF score, sensitivity and specificity were calculated for each total score. Table 6 shows that a threshold value of > = 5 has the highest proportion of correctly classified cases while still maintaining good sensitivity. Therefore, the LBQ-PHF score was divided into two groups: <5 points corresponding to low risk and > = 5 points corresponding to high risk of having poor LBQ in PHF. The corresponding frequencies with sensitivity and specificity are shown in Table 7.

**Table 6:** Detailed report of sensitivity, specificity and, and proportion of correctly classified cases for determination of a threshold value

Threshold value	Sensitivity, %	Specificity, %	Correctly classified, %
>= 0	100	0	38.32
>= 1	100	28.64	56
>= 2	95.31	40.29	61.38
>= 3	92.19	48.54	65.27
>= 4	83.59	60.19	69.16
>= 5	74.22	75.24	74.85
>= 6	52.34	88.83	74.85
>= 7	18.75	96.12	66.47
> 7	0	100	61.68

**Table 7:** Detailed report of corresponding frequencies with sensitivity and specificity. The LBQ-PHF score is divided into two groups: <5 points corresponds to low risk and > = 5 points corresponds to high risk of having poor local bone quality

			DTI		
			>=1.3	< 1.3	Total
LBQ-PHF-score	low risk	n (%)	155 (75.2)	33 (25.8)	188 (56.3)
	high risk	n (%)	51 (24.8)	95 (74.2)	146 (43.7)
Total		n	206	128	334

Applying the LBQ-PHF score with this threshold value to an external test cohort of 107 cases resulted in a sensitivity of 79.2% and a specificity of 86.7% (Table 8).

**Table 8:** Detailed report of corresponding frequencies with sensitivity and specificity after applying the LBQ-PHF score to an external test cohort of 107 cases resulting in a sensitivity of 79.2% and a specificity of 86.7%

			DTI			
			>=1.3	< 1.3		Total
LBQ-PHF-score	low risk	n (%)	72 (86.7)	5 (20.8)		77 (72)
	high risk	n (%)	11 (13.3)	19 (79.2)		30 (28)
Total		n	83	24		107

## Discussion

### Principal findings

Contrary to previous study findings, this is the first study that demonstrates a relationship between fracture pattern, in addition to age, and LBQ in PHF. This finding is revealed by a significant association between the DTI and the morphological Resch classification system as well as additionally measured coronal humeral head alignment.

Mazzucchelli et al. investigated the influence of LBQ measured by DTI on fracture patterns using the Neer classification system and humeral head impaction angle in 191 patients [5]. Besides the observation that neither varus impaction nor any of the Neer fracture types were related to bone quality, they found contrary to our study that valgus impaction significantly depended on good bone quality. This observation was based on a statistical group comparison within the subgroup of valgus impacted PHF of 35 patients with a DTI ≥ 1.4 and 10 patients with a DTI < 1.4 with slight significance (p = 0.047) [5]. The much lower sample size of this subgroup analysis might be one potential reason for the contrary observation concerning coronar humeral head alignment, while we, same as Mazzucchelli et al., did not find any significant association between LBQ and the Neer classification system [5].

Den Teuling et al. investigated in a cohort of 168 consecutive patients the relationship between indicators of osteoporosis measured with the cortical index (CI) and the complexity of fractures of the proximal humerus assessed on plain anteroposterior radiographs using AO/OTA classification system of 2007 [8]. While bone quality seemed to be related to age, no significant differences in the CI were found between simple and complex patterns of PHF [8]. Taskesen et al. observed similar results in a retrospective analysis of 248 patients over 50 years of age with low-energy PHF [7]. While bone quality measured with the DTI was statistically significant different between the sexes and age groups (ages 50–70 and over 70), no difference was observed between the main AO fracture types. Therefore, the authors concluded that osteoporosis might not be the main factor affecting fracture type. However, most of the analyzed fractures were AO type A and B (64% and 32%, respectively), while only 4% were complex type C fractures [7]. Although our study included much more complex fractures, we observed similar results in this respect.

The large sample size of this investigation, inclusion of all age groups, and more comprehensive usage of classification systems compared to previous studies are major strengths of this study. The LBQ-PHF score that emerged out of these findings is easy to use and has solid predictive characteristics with a sensitivity of 79.2% and a specificity of 86.7%. Nevertheless, this scoring system has neither the intention nor the capability to replace the deltoid tuberosity as a current gold standard for LBQ in PHF; however, the LBQ-PHF score might function as an approximation for local bone quality in PHF, for example, in cases of analyses of big data sets and outcome studies with incomplete or non-usable radiographic imaging, where the DTI cannot be calculated.

### Limitations

This study has several other limitations to consider. It was not originally the primary study aim to design a predictive scoring system. This intention arose secondarily out of the primary study results, and must be considered as a major limitation as the study design was not optimal for such purposes. As this scoring system was based solely on chronological age and fracture pattern, modulating factors of bone quality such as secondary diseases were not taken into account. As patient screening was limited solely to coded diagnoses (S42.20-24) according to the International Classification of Diseases − 10th Revision, the proportion of patients diagnosed with osteoporosis is unclear. The study did not include an unfractured control group, and all measurements were performed only by one observer without measurement of intraobserver reliability. Furthermore, although our findings are supported by a large sample size, the distribution of included fracture patterns does not mirror previously reported prevalences. Contrary to several studies, we had many more displaced fractures according to the Neer criteria. This might have been due to several reasons. Firstly, only inpatient cases with both plain radiographs and CT imaging were included in the analyses. Due to national imbursement reasons, inpatient admission and advanced imaging may be biased to cases where the treatment decision-making process is more likely to result in surgical treatment. On the one hand, this selection bias is a major limitation of this study as it may restrict the generalizability of the results to a broader population of PHFs. On the other hand, it could be debated whether it is necessity at all to have a predictive scoring system that includes one-part fractures, as the vast majority of these fractures can be treated nonoperatively.

In addition, this scoring system is based on a threshold value of 1.3 instead of the originally described 1.4 threshold value for the DTI. The original threshold value less than 1.4 for the DTI was calculated through ROC curve analysis using a pQCT cutoff value less than 80 mg/cm^3^, which was a value intended for osteoporosis in the lumbar spine and proximal femur and indicative of low LBQ according to the guidelines of the American College of Radiology [6, 15]; the threshold value of 1.3 is the statistical result of our CART modeling analysis. Nevertheless, both values need to be considered critically as there are no accepted pQCT threshold values for the proximal humerus; therefore, both can function only as an approximation for low LBQ. Furthermore, as this was a retrospective analysis there was no control for correct internally rotated arm position on AP radiograph as described by Spross et al. [6].

Finally, PHF in all age groups were included in our study, because a part of our primary study aim was to investigate the relationship between age and LBQ. A potential disadvantage of this strategy is a heterogeneous cohort that includes younger patients as well as geriatric patients. However, our cohort included predominantly geriatric patients with mean age of 72 years (18.6% below the age of 60 years), reflective of the general epidemiology of this fracture. Ideally, classification/scoring system should have prognostic characteristics. For example, that some types of fractures with low bone quality are at higher risk for impaired outcome after certain treatment modalities. Spross et al. have retrospectively assessed the role of local bone quality using the DTI on the intraoperative reduction result of PHFs and the early cutout rate after open reduction and internal angular stable fixation [16]. They found that besides a long metaphyseal head extension also good bone quality and allows sufficient intraoperative reduction. Furthermore, they observed that good bone quality, a younger age, and a good intraoperative reduction prevented early fixation failure [16]. This is why, local bone quality measured by the DTI might be a relevant factor in treatment algorithm for PHFs [4, 16]. Whether these properties also apply to this scoring system and if it has a clinical relevance remain completely uncertain. In particular, due to the contrary observations compared to previous studies [5, 16], our results have to be considered carefully.

## Conclusion

Age and coronal humeral head alignment are independent predictors of LBQ in PHF. We have developed and validated a simple scoring system based on these variables to assess BQ with solid predictive characteristics. Further investigations are needed to ensure the clinical relevance of this novel score in the management of PHF.

## Data Availability

The datasets used and/or analyzed during the current study are available from the corresponding author on reasonable request.
